# Significant therapeutic effectiveness of durvalumab after chemoradiotherapy for a patient with post‐operative recurrent pulmonary pleomorphic carcinoma

**DOI:** 10.1002/rcr2.781

**Published:** 2021-05-11

**Authors:** So Shimamura, Masafumi Saiki, Shuichiro Ide, Kazuki Masuda, Yoshinori Uchida, Yusuke Sogami, Kazunari Kasai, Tomohiro Inoue, Hiroshi Ishihara

**Affiliations:** ^1^ Department of Internal Medicine II, Faculty of Medicine University of Yamanashi Chuo Japan; ^2^ Department of Pathology, Faculty of Medicine University of Yamanashi Chuo Japan

**Keywords:** Chemoradiotherapy, durvalumab, post‐operative recurrence, programmed cell death‐ligand 1, pulmonary pleomorphic carcinoma

## Abstract

Pulmonary pleomorphic carcinoma (PPC) is a poorly differentiated non‐small cell lung cancer. Because of its rarity, no standard therapy has been established for advanced disease. We herein report on a 62‐year‐old man with recurrent post‐operative PPC, for whom durvalumab after chemoradiotherapy was effective. He was referred to our hospital because of an abnormal shadow in the right upper lung on chest X‐ray. After surgical resection was performed, the imaging and histopathological findings revealed PPC (T4N0M0, stage IIIA) with elevated expression of programmed cell death‐ligand 1 (PD‐L1). A metastasis was found in the left hemithorax 22 months later, and chemoradiotherapy consisting of 60 Gy of radiation and cisplatin plus tegafur/gimeracil/oteracil potassium was administered. Durvalumab was then begun as consolidation therapy. The efficacy of the treatments has continued for longer than 10 months. This case suggests that multidisciplinary treatment with chemoradiotherapy and consolidation immunotherapy may improve the prognosis of locally advanced PPC.

## Graphical abstract

Pulmonary pleomorphic carcinoma (PPC) is a poorly differentiated non‐small cell lung cancer. No standard therapy has been established for advanced disease. We herein report on a 62‐year‐old man with recurrent post‐operative PPC, for whom durvalumab after chemoradiotherapy was effective.

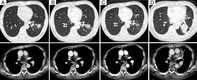

## Introduction

Pulmonary pleomorphic carcinoma (PPC) is a poorly differentiated non‐small cell lung cancer (NSCLC) and is a rare disease that only accounts for 0.4% of all lung tumours [1]. Surgery is selected for early cases, but post‐operative recurrence is common. The effect of cytotoxic chemotherapy is also limited. As it is a rare tumour, no standard therapy has been established. Here, we report a case of recurrent post‐operative PPC, for which durvalumab after concurrent chemoradiotherapy was effective.

## Case Report

**Figure 1:**
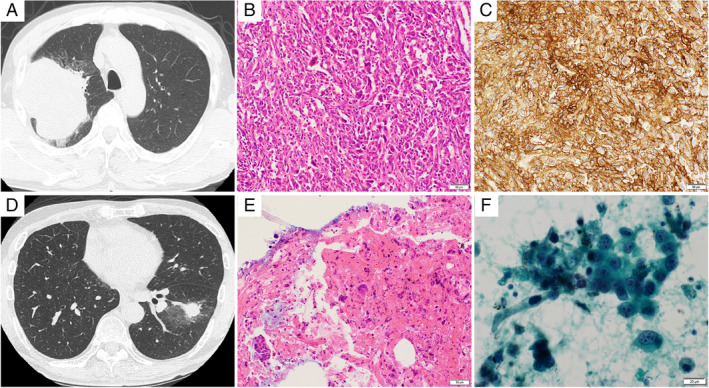
(A) Chest computed tomography (CT) scan showing a tumour in the right upper lobe. (B) Histopathological preparation showing a pleomorphic carcinoma with spindle and giant cells. Haematoxylin and eosin staining, 200× magnification. (C) The programmed cell death‐ligand 1 (PD‐L1) expression was greater than 95%, 200× magnification. (D) One year and 10 months after surgery, a CT scan shows a 20 × 12‐mm mass in the left lower lobe. (E) Histopathological preparation showing atypical cells with a high nuclear–cytoplasmic ratio, 400× magnification. (F) Cytopathological preparation showing large or spindle‐shaped tumour cells with enlarged nuclei, 600× magnification.

**Figure 2:**
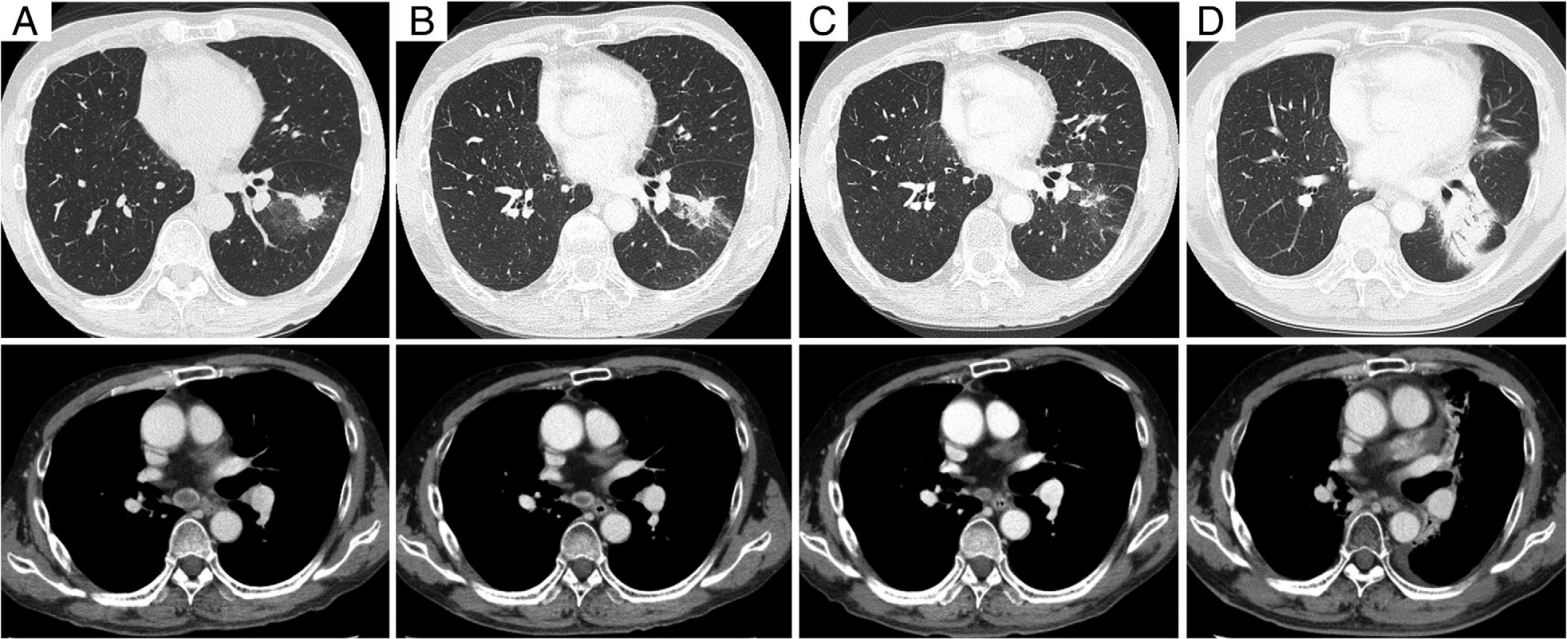
Imaging findings of chest computed tomography (CT) scans over time. (A) Before chemoradiotherapy; (B) at the end of chemoradiotherapy; (C) after the second course of durvalumab, when the size of the lung mass had decreased dramatically; and (D) after the 12th course of durvalumab, when grade I radiation pneumonitis appeared, while the mediastinal lymph nodes were still shrinking.

A 62‐year‐old man was referred to our hospital because of an abnormal shadow in his right upper lung on chest X‐ray. He had neither subjective symptoms nor a clinically significant medical history. He was an ex‐smoker with a Brinkman index of 800. As the lesion was localized to the right upper lobe, he underwent sleeve resection of the right upper lobe and combined resection of the chest wall in February 2018. The imaging and histopathological findings revealed PPC (T4N0M0, stage IIIA). No actionable driver mutations were found, but the tumour proportion score for programmed cell death‐ligand 1 (PD‐L1) expression was greater than 95%. Because the efficacy of cytotoxic chemotherapy for PPC is less than its efficacy for other non‐small lung cancer, with the patient's consent, we did not provide post‐operative adjuvant chemotherapy. During the post‐operative follow‐up, chest computed tomography in December 2019 revealed a 20 × 12‐mm shadow in the left lower lobe and enlarged left hilar and mediastinal lymph nodes. Examination of a transbronchial biopsy specimen revealed histopathological findings of PPC that were similar to that of the resected tumour (Fig. 1). Recurrent cancer of the original histological type that is diagnosed within two years of the initial diagnosis is usually regarded as metastatic disease [2]. Our multidisciplinary team concluded that this patient developed a post‐operative recurrence of pleomorphic carcinoma. Although post‐operative recurrent lung cancer is usually treated with systemic chemotherapy, the beneficial effects of cytotoxic chemotherapy are limited for PPC. There have been occasional reports on the effectiveness of immune checkpoint inhibitor (ICI) for advanced PPC and the effectiveness of chemoradiotherapy for locally advanced PPC [3, 4, 5, 6, 7, 8]. As the patient's lesion was localized, we decided to administer chemoradiotherapy followed by consolidation immunotherapy. In January 2020, the patient started chemoradiotherapy (60 Gy and cisplatin + tegafur/gimeracil/oteracil potassium). At the end of chemoradiotherapy, he showed a partial response (PR), and consolidation therapy with durvalumab was initiated. After two cycles of durvalumab, the diameter of the tumour was smaller than at the initiation of durvalumab. Grade I radiation pneumonitis appeared six months after the initiation of chemoradiotherapy, but did not interfere with the durvalumab treatment. There were no other treatment‐related adverse events. At the time of this report, durvalumab has been administered for more than 10 months, with a durable therapeutic response (Fig. 2). Based on the PACIFIC study, we will complete the administration of durvalumab in 12 months and carry out careful follow‐up.

## Discussion

We reported a case of PPC recurring after surgery, for which consolidation therapy with durvalumab, an anti‐PD‐L1 monoclonal antibody, was effective after chemoradiotherapy.

Surgical resection is the preferred treatment for early‐stage PPC, although it commonly relapses shortly thereafter. Ito at el. reported that out of 15 patients who underwent surgery, six patients, including five with pN0 disease, relapsed after surgery [9]. The effect of cytotoxic chemotherapy for advanced PPC is also limited. Bae et al. reported that for cases that recur after surgery or advanced cases treated with cytotoxic anticancer drugs, the response rate was 0%, and the median overall survival was about five months [10].

However, some reports have suggested that platinum‐based chemoradiotherapy has shown improved results for locally advanced non‐resectable PPC. A case series of 17 patients with PPC reported by Kaira et al. showed that out of four stage III patients, two patients who were treated with chemoradiotherapy achieved PR, with overall survival rates of 27.0 and 12.0 months [3]. Another case series from Tamura et al. of patients with locally advanced or recurring PPC after surgery showed that three patients treated by chemoradiotherapy who used cisplatin + vinorelbine obtained a PR. One of the three survived without progression for longer than five years [4].

An increasing number of recent case reports have shown the effectiveness of ICIs for advanced PPC or PPC recurring after surgery, which might be attributed to the elevated levels of PD‐L1 expression in PPC [5, 6, 7, 8]. Meanwhile, consolidation therapy with durvalumab after chemoradiotherapy has been shown to prolong the survival of patients with locally advanced NSCLC [11]. Although the effectiveness of ICI monotherapy for advanced PPC remains anecdotal, and the effectiveness of chemoradiotherapy for locally advanced PPC is not yet established, the use of chemoradiotherapy plus consolidation durvalumab for locally advanced PPC seems logical. Indeed, a case of PPC was reported in which durvalumab consolidation therapy after chemoradiation had been effectively continued for longer than one year [12].

Our case provides another example of a patient with PPC who was effectively treated by chemoradiotherapy plus consolidation durvalumab. Additional cases and randomized clinical trials are needed to establish the significance of this multimodality therapy for locally advanced PPC.

### Disclosure Statement

Appropriate written informed consent was obtained for publication of this case report and the accompanying images.

### Author Contribution Statement

So Shimamura wrote the manuscript. Shuichiro Ide, Yoshinori Uchida, Kazunari Kasai, and Tomohiro Inoue were involved in interpretation of the data. Kazuki Masuda and Yusuke Sogami were involved in analysis of the data. Masafumi Saiki and Hiroshi Ishihara provided expertise and feedback. All authors drafted the article, revised it critically for important intellectual content, and approved the final version to be submitted.

## References

[1] Hong JY , Choi MK , Uhm JE , et al. 2009. The role of palliative chemotherapy for advanced pulmonary pleomorphic carcinoma. Med. Oncol. 26:287–291. 18989796 10.1007/s12032-008-9117-4

[2] Kozower BD , Larner JM , Detterbeck FC , et al. 2013. Special treatment issues in non‐small cell lung cancer: diagnosis and management of lung cancer, 3rd ed: American College of Chest Physicians evidence‐based clinical practice guidelines. Chest 143:e369–e399. 10.1378/chest.12-2362 23649447

[3] Kaira K , Horie Y , Ayabe E , et al. 2010. Pulmonary pleomorphic carcinoma a clinicopathological study including EGFR mutation analysis. J. Thorac. Oncol. 5:460–465. 20107421 10.1097/JTO.0b013e3181ce3e3c

[4] Tamura Y , Fujiwara Y , Yamamoto N , et al. 2015. Retrospective analysis of the efficacy of chemotherapy and molecular targeted therapy for advanced pulmonary pleomorphic carcinoma. BMC. Res. Notes 8:800. 26682906 10.1186/s13104-015-1762-z PMC4684621

[5] Kanazu M , Uenami T , Yano Y , et al. 2017. Case series of pleomorphic carcinomas of the lung treated with nivolumab. Thorac. Cancer 8:724–728. 28881488 10.1111/1759-7714.12505 PMC5668504

[6] Fujimoto E , Yokoi T , Mikami K , et al. 2018. Successful treatment of pulmonary pleomorphic carcinoma with nivolumab: a case report. Case Rep. Oncol. 11:336–340. 29928213 10.1159/000489392 PMC6006649

[7] Senoo S , Ninomiya T , Makimoto G , et al. 2019. Rapid and long‐term response of pulmonary pleomorphic carcinoma to nivolumab. Intern. Med. 58:985–989. 30568113 10.2169/internalmedicine.0890-18 PMC6478974

[8] Yaguchi D , Ichikawa M , Ito M , et al. 2019. Dramatic response to nivolumab after local radiotherapy in pulmonary pleomorphic carcinoma with rapid progressive post‐surgical recurrence. Thorac. Cancer 10:1263–1266. 30860657 10.1111/1759-7714.13029 PMC6500956

[9] Ito K , Oizumi S , Fukumoto S , et al. 2010. Clinical characteristics of pleomorphic carcinoma of the lung. Lung Cancer 68:204–210. 19577320 10.1016/j.lungcan.2009.06.002

[10] Bae HM , Min HS , Lee SH , et al. 2007. Palliative chemotherapy for pulmonary pleomorphic carcinoma. Lung Cancer 58:112–115. 17574296 10.1016/j.lungcan.2007.05.006

[11] Antonia SJ , Villegas A , Daniel D , et al. 2017. Durvalumab after chemoradiotherapy in stage III non‐small‐cell lung cancer. N. Engl. J. Med. 377:1919–1929. 28885881 10.1056/NEJMoa1709937

[12] Yorozuya T , Taya T , Yasuda K , et al. 2020. Long‐term response with durvalumab after chemoradiotherapy for pulmonary pleomorphic carcinoma: a case report. Thorac. Cancer 11:1090–1093. 32045109 10.1111/1759-7714.13331 PMC7113037

